# Cannabinoid Receptors as Target for Treatment of Osteoporosis: A Tale of Two Therapies

**DOI:** 10.2174/157015910792246173

**Published:** 2010-09

**Authors:** Aymen I Idris

**Affiliations:** Bone Research Group, Molecular Medicine Centre, University of Edinburgh, Western General Hospital, Edinburgh, EH4 2XU, UK

**Keywords:** Cannabinoid, osteoporosis, bone, anti-resorptive, anabolic, Rimonabant^©^, CB1, CB2, GPR55.

## Abstract

The central nervous system plays an important role in regulating bone metabolism in health and in disease with a number of neurotransmitters been reported to influence bone cell activity through a central relay. In keeping with this, recent studies demonstrated that endocannabinoids and their receptors are involved in the pathogenesis of osteoporosis. The endocannabinoids anandamide and 2-arachidonylglycerol are found in the skeleton and numerous studies also showed that bone cells express the cannabinoid receptors CB1 and CB2 and the orphan receptor GPR55. Pharmacological and genetic inactivation of CB1, CB2 and GPR55 in adult mice suppress bone resorption, increase bone mass and protect against bone loss, suggesting that inverse agonists/antagonists of these receptors may serve as anti-resorptive agents. In the ageing skeleton however CB1 and CB2 receptors have a protective effect against age-dependent bone loss in both male and female mice. CB1 receptor deficiency in aged mice results in accelerated age-dependent osteoporosis due to marked increase in bone resorption and significant reduction in bone formation coupled to enhanced adipocyte accumulation in the bone marrow compartment. Similar acceleration of bone loss was also reported in CB2 deficient mice of similar age but found to be associated with enhanced bone turnover. This review summarises *in vitro* and *in vivo* findings relating to the influence of cannabinoid ligands on bone metabolism and argues in favour of the exploitation of cannabinoid receptors as targets for both anabolic and anti-resorptive therapy for treatment of complex multifaceted bone diseases such as osteoporosis.

## INTRODUCTION

The endocannabinoid system is a complex network of endogenous ligands, membrane receptors and metabolising enzymes (reviewed in [1]). Cannabinoids exert a plethora of pharmacological responses in mammalian cells and their receptors are known to be involved in the regulation of numerous physiological processes including neurotransmission, pain perception, learning, memory, cardiovascular homeostasis, appetite, motor function and the immune response (reviewed in [1-3]). The endocannabinoid ligands anandamide (AEA) and 2-arachidonylglycerol (2-AG) are responsible for most pharmacological actions associated with cannabinoid receptors in mammalian cells (reviewed in [4]). AEA and 2-AG are highly expressed in the brain and are also detected in a number of peripheral tissues including heart, liver, kidney, testis and blood [5-12]. Cannabinoid receptors are also activated by plant derived cannabinoids (phytocannabinoids) such as Δ^9^-tetrahydrocannabinol (Δ^9^-THC) and a number of synthetic non-classical cannabinoids such as CP55,940, JWH133 and HU308 [4]. A number of synthetic compounds including SR141716A (also known as Rimonabant^©^), AM251 and AM630 are described as inverse agonists/antagonists due to their ability to down regulate the activity of cannabinoid receptors in the presence and absence of agonist binding [13-21]. Endocannabinoids and their synthetic analogues bind and activate two known cannabinoid receptors: CB1 and CB2, both of which are members of the G-protein coupled receptor family [22, 23]. CB1 and CB2 receptors are coupled to adenylyl cyclase and cyclic adenosine monophosphate (cAMP) together with a number of other second messengers including phospholipase C, PI3 Kinase/Akt and ceramide synthesis [24-28]. Cannabinoid type 1 receptors (CB1) are mainly expressed in the brain, whereas cannabinoid type 2 receptors (CB2) are found in the periphery predominantly on cells of the immune system [29, 30]. However, recent studies have reported that a number of other tissues, organs and cells including bone cells and adipocytes also express CB1 and CB2 receptors [31, 32]. Recent findings also suggest that the “orphan” G protein-coupled receptor GPR55 might represent a third cannabinoid receptor [33, 34]. GPR55 is predominately expressed in the brain but also found in peripheral tissues such as spleen [35]. Cannabinoid ligands in particular AEA are also known to activate other targets such as the ligand gated transient receptor potential vanilloid type 1 receptor (TRPV1) [36].

Endocannabinoids and their receptors influence bone cell differentiation, survival and function. Identification by us and others of the role of CB1 and CB2 receptors in bone mass suggests that pharmacological modulation of these receptors are capable of suppressing excessive bone loss, a hallmark of a variety of bone diseases including osteoporosis. Recently, it became apparent that other receptors and channels closely related to the endocannabinoid system – namely TRPV1 and GPR55 - are also implicated on the regulation of bone cell activity and bone mass. Together with earlier findings, these studies consolidate the role of the skeletal endocannabinoid system as a regulator of bone remodelling and pave the way for identification of diverse novel therapeutic strategies through which it might be possible to modulate cannabinoid receptors and derive future treatments for bone disorders.

### Bone Remodelling

**Fig. (1):**
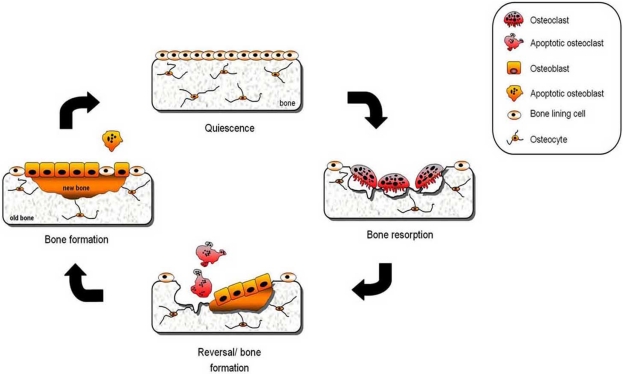
**The bone remodelling cycle**. Upon stimulus, embedded osteocytes within the bone matrix attract osteoclasts and their precursors to the remodelling site. Mature osteoclasts first attach to bone surface and then resorb the bone matrix (bone resorption). Following resorption and osteoclast apoptosis, there is a reversal phase during which osteoblast recruitment and proliferation occurs. Fully differentiated osteoblast deposit osteoid on the resorption site thereby initiating bone formation. Bone formation is followed by a phase during which freshly laid osteoid becomes mineralised and covered by bone lining cells.

Bone is a cell-rich, metabolically active and specialised connective tissue that is continuously undergoing a process of renewal and repair known as “bone remodelling”. Bone remodelling is highly coordinated by various hormones, cytokines, and peptides and is divided into four stages, bone resorption, the reversal phase, bone formation and the quiescence phase (Fig. **1**). Bone resorption is the process by which old bone is removed by the osteoclast; a large, multinucleated, motile and highly specialised cell of haematopoietic origin [37]. Following chemical stimuli, micro-damage or mechanical stress, mature osteoclasts and their precursors migrate to the site which is to be resorbed by a mechanism not yet fully understood. Several investigators have proposed that embedded osteocytes within the bone matrix sense the need for remodelling and instruct the bone forming osteoblasts to secrete collagenases that remove unmineralised matrix and direct mature osteoclasts and their precursors to the remodelling site [38, 39]. Osteoclast formation and activity is controlled by the combined action of receptor activator of nuclear factor kappa-B ligand (RANKL) [40], osteoprotegerin (OPG) [41] and monocyte colony forming factor (M-CSF) [42] produced by cells of the osteoblastic lineage. RANKL and M-CSF represent the minimal essential stimulatory cytokines required for osteoclast formation under normal conditions whereas OPG is inhibitory [41]. The amount of bone resorbed is dictated by the number, size and the life span of newly formed osteoclasts that depended entirely on the level of local and systemic factors such as 1, 25-(OH)_2_ vitamin D_3_ (VD3) and parathyroid hormone (PTH) [43, 44]. Mature osteoclasts undergo rapid apoptosis and are rapidly removed by phagocytes thereby signalling the reversal phase that represents the intermediate period after resorption has ceased and before bone formation begins (Reviewed in [45]).

Fresh bone is laid down by the mononucleated osteoblasts that originate from mesenchymal osteoprogenitor cells found in the bone marrow (BM) [46]. Mesenchymal osteoprogenitors are multipotent cells which can also differentiate into various cell types including adipocytes and chondrocytes [46]. Bone formation is initiated with the attraction of osteoblast precursors to the freshly resorbed site by the chemotactic transforming growth factor β (TGFβ) and bone matrix proteins such as type-1 collagen, which are both released during the resorption process [47, 48]. A number of systemic hormones including PTH, oestrogen and VD_3_ are also known to stimulate the differentiation of precursor cells into mature osteoblast capable of matrix synthesis and bone formation [46]. The average lifespan of human osteoblasts is three month, after which approximately 65% of functioning osteoblasts undergo apoptosis [49]. The remaining mature osteoblasts are either buried within the newly deposited matrix as osteocytes or converted to lining cells that cover the majority of quiescent bone [50].

### Abnormal Bone Remodelling and its Relation to Osteoporosis

Imbalance in bone formation and bone resorption caused predominately by changes in local and systemic factors is the major common cause of most - if not all - bone disorders. Osteoporosis is a metabolic disorder characterised by disparity in osteoblast and osteoclast activity leading to gradual deterioration of bone mass and enhanced bone fragility and fracture risk (reviewed in [51]). Osteoporotic fractures represent one of the main causes of morbidity among elderly patients across the developing world [52-60]. The most common causes of osteoporosis are oestrogen deficiency and glucocorticoid treatment both of which are associated with enhanced bone resorption coupled to a significant reduction in bone formation [51]. In postmenopausal osteoporosis, oestrogen deficiency is associated with two distinct phases of bone loss characterised by different cellular pattern and behaviour [61]. The acute phase of bone loss that immediately follows oestrogen withdrawal is characterised by excessive bone resorption. This is caused by rapid rise in osteoclast number that is coupled to a marked increase in osteocyte and osteoblast apoptosis [62, 63]. At the molecular level, sudden drop in oestrogen is associated with raised levels of RANKL and M-CSF coupled to a significant drop in the production of inhibitory factors such as OPG [64]. This phase is followed by a long lasting period of sustained age-dependent bone loss due to a significant reduction in osteoblast differentiation and bone formation coupled to marked increase in adipocyte differentiation [65-67]. Long-term treatment with glucocorticoids is the most common cause of secondary osteoporosis (reviewed in [68]). At the cellular level, glucocorticoids regulate both bone formation and resorption by inhibiting osteoblast differentiation and reducing the production of factors that enhance osteoclast formation such as RANKL [68, 69]. A number of other skeletal disorders such as rheumatoid arthritis (reviewed in [70, 71]) and cancer associated bone diseases (reviewed in [72]) are also characterised by excessive bone resorption leading to bone loss. 

## NEURONAL MEDIATORS OF BONE REMODELLING 

The central nervous system (CNS) plays an important role in regulating bone remodelling, with a number of neurotransmitters and systemic hormones been reported to influence bone mass through a central relay (Table ** 1**) [73, 74]. For example, glutamate and N-methyl-d-aspartate receptors are present on osteoblasts and osteoclasts and regulate bone turnover by stimulating osteoblast differentiation and function (Reviewed in [75]). Nitric oxide is known to play a role in regulating bone remodelling at the local level [76], but recent studies showed that mice lacking neuronal nitric oxide synthase display high bone mass due to low bone turnover [77]. Pituitary-derived hormones such as thyroid and follicle stimulating hormones are also involved in the regulation of bone remodelling by influencing osteoclast and osteoblast differentiation [78, 79]. Bearing in mind that mammalian bones are widely innervated by sympathetic and sensory nerves [80-83] and that activation of the sympathetic nervous system (SNS) is known to regulate bone formation and resorption [84, 85], it is reasonable to suggest that a number of these factors may regulate bone turnover through a central relay. 

**Fig. (2):**
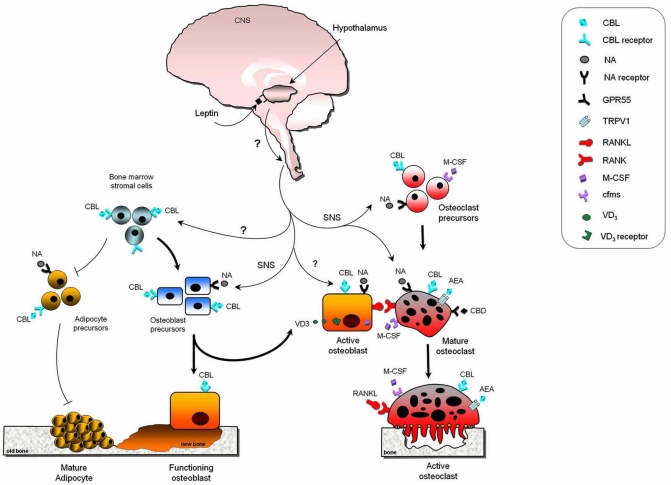
Schematic illustration of the current model of local and systemic regulation of bone cell differentiation and function by cannabinoid ligands. Leptin regulates bone mass through a neuronal hypothalamic relay involving β-adrenergic neurones and endocannabinoid system within the sympathetic nervous system (SNS). Noradrenaline is known to increase bone loss by stimulating osteoclast formation and resorption. Cannabinoid receptors are likely to influence the hypothalamic action of leptin on bone formation by a central relay. Moreover, mature osteoblasts express CB1 and CB2 receptors and secrete AEA and 2-AG that in turn enhance RANKL induced osteoclast formation, thereby influencing osteoblast-osteoclast coupling. Cannabinoid ligands (CBL) are also involved in the regulation of osteoclast survival, polarisation and activity by acting on CB1 and CB2 receptors expressed on mature osteoclasts. CBL are capable of regulating bone formation by either directly acting on CB1 and CB2 receptors on osteoblasts or indirectly by inhibiting the production of the catecholamine noradrenaline, an inhibitor of osteoblast differentiation. Acting on CB1 receptors expressed on BM stromal cells, cannabinoid receptor agonists stimulate osteoblast differentiation and inhibit adipocyte accumulation in the bone marrow. TRPV1 and GPR55 expressed by osteoblasts and osteoclasts are likely to be responsible for some of the skeletal action of AEA and other cannabinoid ligands. Abbreviation: SNS - sympathetic nervous system; CB – cannabinoid; RANKL - receptor activator of NFκ B ligand; VD3 – vitamin D3, M-CSF - macrophage colony stimulating factor; cfms - M-CSF receptor; NA – noreadrenaline; AEA – anandamide; CBD – cannabidiol. Question mark (?) denotes unknown factors.

Neuropeptide Y (NPY) is expressed widely in the central and peripheral nervous systems. Studies showed that specific deletion of the hypothalamic NPY receptors results in a high bone mass phenotype due to enhanced osteoblast differentiation and bone formation [86], confirming the involvement of the CNS in the regulation of bone growth. Recently, studies examining the role of the adipocyte-derived hormone leptin in bone remodelling, made substantial advances in our understanding of the mechanisms by which the central and peripheral nervous systems control bone remodelling (Reviewed in [73, 74]). Takeda *et al*. showed that leptin reduced bone formation and mass through a neuronal hypothalamic relay involving inhibition of β-adrenergic neurones within the sympathetic nervous system (Fig. **2**) [87, 88]. Later work by the same group added that beta-2-adrenoreceptor indirectly influences osteoclastic bone resorption by regulating expression of RANKL on osteoblasts [89]. We have recently found evidence that pharmacological activation of β-adrenergic receptors can also stimulate osteoclast formation directly by acting on osteoclast precursors indicating that β-adrenergic receptors can directly and indirectly modulate osteoclast formation and function (Fig. ** 2**) [90]. These studies together provided evidence for the notion of so-called neurogenic relay which controls bone turnover and also encouraged research into uncovering the role of other neurotransmitters on bone remodelling. 

## THE SKELETAL ENDOCANNABINOID SYSTEM

### Cannabinoids and their Receptor Expression in Bone 

A number of recent studies reported that endocannabinoids and their metabolising enzymes are present in the skeleton. AEA and 2-AG are present in the bone marrow and within the metabolically-active trabecular compartment, at levels in the same magnitude as the brain [12, 91]. Both osteoblasts and osteoclasts are capable of producing AEA and 2-AG in culture [12, 91, 92]. Complementary to these findings, a number of cell types within the bone micro-environment including osteoblasts, osteoclasts, osteocytes, stromal cells and adipocytes are found to express the endocannabinoid metabolising enzymes NAPE-phospholipase D, fatty acid amide hydrolyse, diacylglycerol lipases and monoacylgycerol lipase (our unpublished data; [12, 93]). The cannabinoid receptors CB1 and CB2 and a number of closely related receptors and channels such as GPR55 and TRPV1 are found in the skeleton. CB1 receptors are known to be expressed on nerve fibres intervening bone [12, 94] and on cells of the immune system within the BM compartment [2, 30]. We and others reported that CB1 receptors are also detected on osteoblasts, osteoclasts and BM derived adipocytes at both protein and mRNA levels [95, 96]. CB2 receptors on the other hand are highly expressed on peripheral blood mononucleated cells and immune cells including macrophage, monocytes, B and T lymphocytes [26, 30, 97-100]. Osteoblasts, osteoclasts and osteocytes also express CB2 receptors at significantly higher level than that reported for CB1 [31, 32, 93, 96]. Recent studies reported that bone cells also express GPR55 and TRPV1 which are known to be targeted by endocannabinoids and synthetic cannabinoid ligands [36, 96, 101, 102, 111].

### Cannabinoid Inverse Agonists/Antagonists as AntiResorptive Agents

The prevention and treatment of excessive bone resorption is based on the use of anti-resorptive agents such as Bisphosphonates and calcitonin. Anti-resorptive drugs are a class of therapeutic agents that selectively/specifically target and inhibit osteoclast differentiation and function with minimal direct activity toward osteoblasts (Reviewed in [103, 104]). We have found that CB1 and CB2 expression on osteoclast and their BM precursor cells is highly up regulated in ageing mice and following oestrogen deficiency in adult mice [95]. To determine the relevance of this finding, we studied the effects of CB1 receptor inactivation on bone loss in ovariectomised mice, a well established model of acute bone loss following oestrogen deficiency [105]. We reported that mice lacking CB1 receptors are protected from ovariectomy-induced bone loss and exhibited reduced osteoclast number and bone resorption in comparison to wild type littermates [32]. We also showed that CB1 deficiency in healthy mice results in accelerated bone growth in neonate and high bone mass in adult mice due to reduced osteoclast number and bone resorption [32]. Surprisingly, the number of osteoblasts and all parameters of bone formation remains unaffected by CB1 deficiency during growth and early adulthood [32, 95]. In contrast, CB2 deficient mice of similar age showed no significant changes in bone mass [31, 106]. Based on these findings, it is clear that CB1 receptors regulate osteoclastic bone resorption in adult mice and that under conditions of increased bone turnover these receptors may regulate bone loss. Interestingly, recent studies have reported that adult mice deficient in the orphan receptor GPR55 display increased peak bone mass due to a significant defect on osteoclastogenesis but the number of osteoblasts remains unaffected [107]. The skeletal abnormalities reported in GPR55 KO mice were remarkably similar to those observed in CB1 deficient mice [32]. Bearing in mind that GPR55 is activated by a number of cannabinoids ligands including endocannabinoids and the CB1 selective agonist AM251 [108, 109], it is likely that GPR55 is involved in the regulation of endocannabinoids action in osteoclastic bone resorption. 

**Fig. (3):**
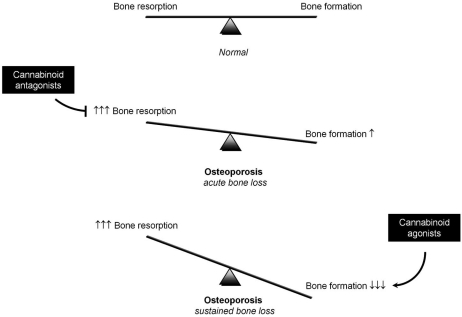
**Hypothetical model for prevention and treatment of postmenopausal osteoporosis using cannabinoid ligands.** Cannabinoid receptors play a role in regulating osteoclast and osteoblast differentiation and activity in the ageing skeleton. Osteoblast and osteoclast activity are balanced during skeletal growth and early adulthood. Following oestrogen deficiency after menopause, acute bone loss occurs due to a significant rise in osteoclast number. During this phase, cannabinoid receptor inverse agonists/antagonists may prevent excessive bone loss by reducing osteoclast number and bone resorption. Activation of cannabinoid receptors using cannabinoid agonist may restore bone loss incurred during the prolonged phase of bone loss by promoting osteoblast differentiation and bone formation.

Over recent years, we have extensively tested whether pharmacological blockage of cannabinoid receptors may be of value in the prevention of acute bone loss. In our studies, we demonstrated that treatment with the CB1 selective inverse agonist/antagonist AM251 and the CB2 selective inverse agonist/antagonist AM630 reduced osteoclast number and bone resorption *in vivo *and protected against ovariectomy induced bone loss in adult mice [32, 95]. Other workers reported that the novel CB2 selective antagonist Sch.036 prevented inflammation and bone damage in arthritic mice [110]. Interestingly, genetic inactivation of CB2 receptors in adult mice only partially protected from bone loss due to ovariectomy [106]. This suggests that prevention of bone loss following treatment with CB2 selective inverse agonists/antagonists such as AM630 and Sch.036 may occur at least in part by an effect on CB1 receptors. Nevertheless, these findings together confirm the anti-resorptive capabilities of cannabinoid receptor - in particular CB1 - blockage in animal models of acute bone loss (Fig. **3**).

A number of *in vitro* studies have recently shed light on the mechanisms by which cannabinoid receptor blockage regulate osteoclastogenesis. For example, the CB1 selective inverse agonists/antagonists AM251 and Rimonabant^© ^and the CB2 selective inverse agonist/antagonist AM630 are capable of exerting direct inhibitory effects on osteoclast formation, fusion, polarisation and activity [32]. Recent studies in our laboratories demonstrated that cannabinoid receptors also regulate osteoclastogenesis by indirectly influencing “osteoblast-osteoclast coupling” (Fig. **2**). For example, we showed that osteoclast formation is significantly reduced in osteoblast – bone marrow co-cultures in which the osteoblasts were prepared from CB1KO mice [95, 106]. Further studies showed that osteoblast cultures generated from CB1KO mice express less RANKL therefore confirming the reduced capabilities of these osteoblast to support osteoclast formation normally [95]. Cannabinoid receptor activation using the endocannabinoids AEA and 2-AG, CB1/2 synthetic agonist CP55,940 and CB2 selective agonist JWH133 and HU308 enhance osteoclast number, increase osteoclast size and multinuclearity and stimulate bone resorption [32, 92, 106]. As with CB1 and CB2 selective agonists, TRPV1 and GPR55 receptor agonists are also capable of increasing osteoclast number in human and mouse cultures [96, 107]. A recent study in our laboratories showed that the TRPV1 agonist capsaicin enhances osteoclast formation, whereas the antagonist capsazepine suppressed osteoclast and osteoblast differentiation and function *in vitro* and inhibited ovariectomy induced bone loss in mice by reducing indices of bone resorption and bone formation [111]. These results together with earlier findings reported by Rossi and colleagues [96] clearly demonstrate that pharmacological blockade of TRPV1 ion channels is capable of inhibiting osteoclastic bone resorption and as a result protects against bone loss in animal model of osteoporosis [96, 111]. Bearing in mind that cannabinoid receptors, TRPV1 and GPR55 are known to co-exist in a number of cells including osteoclasts and osteoblasts [107, 112-115], it is possible that some of cannabinoids actions may actually be mediated *via *TRPV1, GPR55 and/or other unknown mechanism(s). In keeping with this, we and others found evidence that activation of CB2 – using the CB2 selective agonists HU308 and ajulemic acid - inhibits osteoclast formation under certain conditions by an unknown mechanism(s) [31, 106, 107, 116]. Regardless of this, it is clear that cannabinoid receptor inverse agonists/antagonists show value as anti-resorptive agents for the prevention of osteoporosis and other bone diseases characterised by increased osteoclast activity (Fig. **3**).

### Endocannabinoids and Synthetic Cannabinoid Agonists as Bone Anabolic Agents

Bone formation plays a critical role in age-related bone loss and the pathogenesis of a number of bone diseases including postmenopausal and drug-induced osteoporosis [51]. In recent years, extensive research into pathways involved in the regulation of osteoblast differentiation and activity has led to the discovery of a number of bone anabolic agents that stimulate bone formation such as exogenous PTH (also known as teriparatide or Forteo©) (Reviewed in [117]). Endocannabinoids and their receptors are involved in the regulation of osteoblast differentiation and bone formation (Fig. **2**). The first evidence supporting a potential effect of cannabinoids on bone formation came from two independent studies examining the role of leptin on food intake and energy metabolism. Ducy *et al.* showed that leptin, acting on the hypothalamus, influences bone remodelling by negatively regulating bone formation [87]. Complementing this finding, Ravinet *et al.* reported that genetic inactivation of CB1 receptors reduces leptin levels and body weight in experimental animals [118]. Together these studies suggest that CB1 receptors influence - at least in part - the effects of leptin in osteoblast activity and bone formation (Fig. **2**). We and others showed that the endocannabinoids AEA and 2-AG, the synthetic CB1/2 agonist CP55,940 and CB2 selective agonists HU308 and JWH133 stimulate early differentiation of BM derived osteoblast precursors and enhance bone nodule formation in osteoblast cultures *in vitro *(Fig. **2**) [31, 93, 119]. Conversely, treatment with the CB receptor inverse agonist/antagonist AM251 suppresses osteoblast number and function acting on CB1 receptors [95, 106, 119]. We and others also showed that BM stromal cells from CB1 and CB2 deficient mice had a significantly reduced capacity to form mineralised bone nodules when cultured in osteogenic medium and had lower expression of the osteoblast specific alkaline phosphatase and core binding factor alpha1 (Cbfa1) [31, 95], indicating that endocannabinoids and their receptors are capable of exerting a cell autonomous effect on osteoblast and their precursors (Fig. **2**).

In most of osteoporotic patients, sustained bone loss is mainly due to significant reduction in osteoblast number and bone formation (Fig. **3**) [65, 67]. It was reported that CB2 causes accelerated age-related osteoporosis due to enhanced bone turnover [31]. In our studies however we found that bone loss in ageing CB2 deficient mice is associated with elevated bone resorption coupled to a significant reduction in osteoblast number and bone formation [93]. In agreement with this, activation of the peripherally abundant CB2 receptors, using JWH133 or HU308, protected against bone loss in ovariectomised mice by increasing bone formation markers [31, 93]. These findings - together with evidence showing strong association of CB2 polymorphisms with osteoporosis in women [120, 121] – suggest that CB2 agonists show promise for the treatment of osteoporosis as stimulator of bone formation (Fig. **3**). However a recent study using the mouse traumatic brain injury model to investigate the role of cannabinoid receptors in bone formation revealed that CB1 – not CB2 – receptor activation is responsible for increased bone formation following brain injury [12]. The authors of this report went on to suggest that activation of CB1 present on presynaptic nerve endings influence new bone formation by suppressing the release of noradrenaline, an inhibitor of osteoblast activity [12, 122]. Taking into account all findings to date, it is clear that cannabinoid receptor - in particular CB1 - activation regulates osteoblast differentiation and function by directly acting on bone cells and/or indirectly influencing the release of systemic mediators of bone formation such as noradrenaline (Fig. **2**). 

Encouraged by these findings, we recently investigated the effects of pharmacological and genetic modulation of CB1 receptors on osteoblast differentiation and function in ageing osteoporotic mice. We reported that CB1 deficiency profoundly worsen osteoporosis in 12 month old female mice and resulted in marked loss of bone in male mice of similar age [95]. Detailed histological analysis in our studies showed that CB1 deficiency at this age was associated with a significant reduction in osteoblast number and bone formation resulting in a significant bone loss despite of the significant reduction in osteoclast number (Fig. **3**) [32, 95]. This has led us to conclude that age-related osteoporosis associated with CB1 deficiency is not due to increased bone resorption, but is instead due to reduced osteoblast differentiation and bone formation. Osteoporosis in CB1 KO mice was also associated with a striking accumulation of adipocytes in the BM compartment [95]. Studies conducted on bone marrow stromal cells (MSC) – a common precursor to adipocyte and osteoblast - revealed that cultures deficient in CB1 receptors showed a significant reduction in osteoblast differentiation mainly due to an increased capacity of MSC to differentiate into adipocytes [95]. This shift in lineage commitment is coupled to a significant down regulation of the osteoblast specific gene Cbfa1 in osteoblasts and upregulation of cAMP response element binding (CREB) phosphorylation in preadipocytes [95]. All these effects were reproduced pharmacologically in wild type cultures by treating with the CB1 selective inverse agonist/antagonist AM251 [95]. However the pharmacological effects of cannabinoid receptor modulation in adipocyte differentiation reported in the literature are difficult to interpret. For example, endocannabinoids are reported to activate the expression of the adipogenic gene peroxisome proliferator-activated receptor gamma (PPARγ), a powerful stimulator of adipocyte differentiation [123, 124]. Conflicting reports showed that the CB1 selective agonist/inverse antagonist Rimonabant^©^ inhibits cell proliferation but increases markers of adipocyte maturation in preadipocyte cultures [125]. In broad agreement with the latter, we showed that treatment with the CB1 selective agonist/inverse antagonist AM251 inhibits stromal cell differentiation but increases adipocyte differentiation and enhances the expression of adipocyte specific genes such as Fatty acid-binding protein 4, Ccaat-enhancer-binding proteins C/EBPβ and C/EBPα ([95] and Idris *et al*. unpublished data). Whilst these findings raise the possibility that long term use of cannabinoid receptor inverse agonists/antagonists may suppress osteoblast differentiation and enhance adipogenesis in the bone marrow, they also provide an explanation for the stimulatory effect of cannabinoid agonists on osteoblast activity and bone formation.

## CONCLUDING REMARKS AND FUTURE PERSPECTIVE

There is a steadily growing body of evidence suggesting that the skeletal endocannabinoid system plays an important role in the regulation of bone mass in health and in disease. Cell and tissue based studies showed that bone cells express cannabinoid receptors and the machinery for the synthesis and breakdown of endocannabinoids, thereby indicating that endocannabinoids influence bone remodelling acting on CB1 and CB2 receptors expressed on bone cells. Expression of CB1 within innervating neurones however raises the possibility that cannabinoids regulate bone mass by a neuronal mechanism. To fully address this issue, future studies should examine the bone phenotype of animals with site specific inactivation/overexpression of cannabinoid receptors. Genetic and pharmacological studies in adult mice, CB1 and CB2 receptor inverse agonists/antagonists show promise as anti-resorptive agents. On the basis of recent reports of depression and suicidal behaviour associated with the use of the CB1 receptor selective inverse agonist Rimonabant^©^, it is clear that the eagerly awaited peripherally-active cannabinoid agents that don’t cross the blood-brain barrier would be of substantial clinical value for treatment of bone diseases. Future studies with such compounds should examine the effects of long term blockage of cannabinoid receptors on the activity of osteoblast and other cells such as osteocytes and adipocytes. Such studies should also establish whether pharmacological action of cannabinoid ligands is mediated *via *CB1/CB2 independent targets such as GPR55 and TRPV1. The role of cannabinoid receptors on osteoblast activity and bone formation is interesting and suggests that endocannabinoids possess bone anabolic capabilities. Of the endogenous cannabinoid ligands discovered to date, only AEA and 2-AG have been investigated in bone. Future studies should therefore assess the bone anabolic capabilities of endocannabinoid structurally-related derivatives such as virodhamine, noladin ether, N-arachidonoyl dopamine and Δ^9^-THC in ageing rodents and most importantly compare their efficiency to that of well-established bone anabolic agents such as PTH. The outcome of such studies will greatly enhance our understanding of the role of the skeletal endocannabinoid system in bone pathologies and encourage the development of cannabinoid-based therapy aimed at providing both anti-resorptive and anabolic effects in bone.

## Figures and Tables

**Table1:** Neuronal Mediators with Reported Influence on Bone Mass and Cell Differentiation and Function

Ligands	Osteoblast	Osteoclast
Glutamate	↑ Osteoblast differentiation	↑ Osteoclast formation
Nitric oxide	↑ Osteoblast differentiation	↑ Osteoclast formation
Leptin	↓ Osteoblast differentiation	↑Osteoclast formation
Neuropeptide Y	↓ Osteoblast differentiation	↔ Osteoclast formation
Thyroid stimulating hormone	↓ Osteoblast differentiation	↓Osteoclast formation
Follicle stimulating hormone	↔ Osteoblast differentiation	↑ Osteoclast formation
Noradrenaline	↓↔ Osteoblast differentiation	↑ Osteoclast formation
Cannabinoid receptor 1	↑ Osteoblast differentiation	↑ Osteoclast formation ↑ Bone resorption
Cannabinoid receptor 2	↑ Osteoblast differentiation	↑↓ Osteoclast formation ↓ Bone resorption
TRPV1 agonists	N/T	↑ Osteoclast formation
GPR55 agonists	↔ Osteoblast differentiation	↑ ↓Osteoclast formation

N/T denote non-tested.

## ABBREVIATIONS

2-AG: = 2-arachidonylglycerol
AEA: = Anandamide
BM: = Bone marrow
C/EBP: = Ccaat-enhancer-binding protein
cAMP: = Cyclic adenosine monophosphate
CB: = Cannabinoid
cAMP: = Cyclic adenosine monophosphate
Cbfa1: = Core binding factor alpha1
CNS: = Central nervous system
Δ^9^-THC: = Δ^9^-tetrahydrocannabinol
DAGL: = Diacylglycerol lipase
FAAH: = Fatty acid amide hydrolase
M-CSF: = Macrophage colony stimulating factor
MSC: = Bone marrow stromal cells
NAPE: = N-arachidonoyl phosphatidylethanolamine
NAPE: = N-arachidonoyl phosphatidylethanolamine
NPY: = Neuropeptide Y
OPG: = Osteoprotegerin
PI3K: = Phosphoinositide 3-kinase
PKA: = Protein Kinase A
PLC: = Phospholipase C
PPARγ: = Peroxisome proliferator-activated receptor gamma
PTH: = Parathyroid hormone
RANKL: = Receptor activator for NFκB ligand
SNS: = Sympathetic nervous system
TBI: = Traumatic brain injury
TGFβ: = Transforming growth factor-beta
TNFα: = Tumour necrosis factor-alpha
TRPV1: = Transient receptor potential vanilloid type 1 receptor
VD3: = 1, 25-(OH)2 vitamin D3
